# Comparison of neutrophil functions in diabetic and healthy subjects with chronic generalized periodontitis

**DOI:** 10.4103/0972-124X.44089

**Published:** 2008

**Authors:** Neetha Shetty, Biju Thomas, Amita Ramesh

**Affiliations:** 1 *Assistant Professor, Department of Periodontics, MCODS, Mangalore, India*; 2 *Professor and Head of Department, Department of Periodontics, ABSMIDS, Mangalore, India*; 3 *Professor, Department of Periodontics, ABSMIDS, Mangalore, India*

**Keywords:** Diabetes mellitus, periodontitis, polymorphonuclear leukocyte

## Abstract

**Background:**

Diabetes mellitus is a systemic condition that has long been associated with an increased risk and severity of periodontal disease. Polymorphonuclear leukocytes (PMNs) play a key role in the maintenance of gingival and periodontal health. Reduced PMN function has been found in patients with diabetes.

**Aim:**

The objective of this study was to evaluate PMN functions in 15 diabetic patients with chronic generalized periodontitis.

**Materials and Methods:**

Chemotaxis, superoxide production, phagocytosis and killing of *Porphyromonas gingivalis* by diabetic PMNs were evaluated relative to healthy and matched controls.

**Results:**

These analyses revealed a significant (*P* < 0.01) depression in the number of diabetic PMNs migrating along an fMLP gradient. In addition, a significant (*P* < 0.01) enhancement of diabetic PMN superoxide production was observed. Phagocytosis (*P* < 0.05) and killing by diabetic PMN of *P. gingivalis* was also impaired significantly (*P* < 0.01).

## INTRODUCTION

Diabetes mellitus (DM) is a major health problem in the world. Approximately 5% of diabetics are classified as type-1 (insulin dependent DM), a condition characterized by abrupt onset at any age, destruction of pancreatic islet cells, and dependence on exogenous insulin. The more prevalent form of diabetes is type-2 (noninsulin dependent DM), a condition which often develops over a period of time, involves reduced responsiveness of tissues to circulating insulin, and is often controlled by diet or oral hypoglycemic agents. Both types are characterized by hyperglycemia, hyperlipidemia, and associated complications.[1,2]

Among the systemic factors, the relationship between periodontal disease and DM has been studied extensively. Many investigators, in their epidemiological, experimental, and clinical studies have reported that the severity of periodontal disease is significantly greater among diabetics than in nondiabetics.[3–8]

Numerous studies have identified a clear role of PMN's in the maintenance of gingival and periodontal health. Reduced PMN function has been found in patients with diabetes. PMN dysfunction studies in diabetics have exhibited defects in chemotaxis, phagocytosis and killing, and increased release of super oxide.[9]

Diabetic patients with chronic periodontitis have depressed chemotaxis compared with nondiabetic patients with chronic periodontitis. Neutrophils from periodontitis patients generate abnormally high levels of oxygen radicals in response to stimuli. This is an important bactericidal mechanism, but the same mechanism has the potential to induce tissue destruction. In diabetic patients with periodontitis, super oxide release is enhanced due to both hyperglycemic state and periodontitis condition which brings about pronounced tissue destruction when compared to healthy subjects with periodontitis.[10,11]

Hence, in the present study, an attempt is made to assess the influence of DM on the neutrophil functions as estimated by chemotaxis, phagocytosis and killing, and super oxide release; and to evaluate the possible correlation of the severity of periodontal disease and neutrophil abnormalities in diabetics and nondiabetics.

## MATERIAL AND METHODS

The subjects for this study were selected from the OPD, Department of Periodontitics, ABSMIDS, and from the diabetic clinic of KSHEMA hospital, Deralakatte, Mangalore.

The study comprised of 60 subjects, inclusive of both sexes, who were in the age group of 30-60 years and were divided into two groups of 30 diabetics and 30 nondiabetics with chronic periodontitis.

Inclusion criteria

1. Number of teeth present - 20.
2. Number of sites involved should have ≥2 mm clinical attachment loss with presence of disease activity as recorded by gingival index (Loe and Silness).

Exclusion criteria

1. History of any systemic disease for the control group.
2. History of any systemic disease other than diabetes for the test group.
3. Brittle diabetics.
4. Pregnant women and lactating mothers.
5. Presence of any habits like smoking and alcoholism.
6. History of antibiotic therapy within 6 months prior to study.

A standard profoma consisting of the following data: name, age, sex, medical and past dental history, plaque index (Silness and Loe), gingival index (Loe and Sillness), and clinical attachment for each patient were recorded. Each patient was examined using a mouth mirror and William's graduated periodontal probe under artificial light.

### Neutrophil function tests

The tests include chemotaxis, phagocytosis and killing, and super oxide estimation.[12] Five milliliters of venous blood was drawn from the antecubital vein with a needle and a disposable syringe; 2.5 ml of this blood was transferred into a plain vial (for phagocytosis), and the remaining 2.5ml into the vial containing EDTA and transported to the laboratory.

### Preparation of cells

After doing a total count and differential count of WBCs to confirm their presence in normal range, the blood in the plain vial was incubated at 37°C for 2 hours and the serum was separated for use in the phagocytic study. The blood collected in the vials with EDTA were mixed with equal quantities of RPMI 1640 and one-third volume of 6% dextran and kept at room temperature for 1 hour. Supernatant was collected in test tubes and washed 3-4 times with RPMI to remove traces of plasma and dextran and cells were maintained in RPMI 1640 medium.[13]

### Neutrophil chemotaxis

The cells were prepared in RPMI 1640 solution and the neutrophils adjusted at 5 × 10^[6]^ /ml, making sure that the cell concentration was same in the test and control samples. The lower compartment of the chemotactic chamber was filled with FMLP as the chemotactic factor (FMLP/mg/ml in Hanks balanced salt solution).

The upper compartment was filled with cell suspension and placed inside the lower compartment, and the chamber was incubated at 37°C in air for 3 hours. At the end of 3 hours, the test was taken out of the incubator and the upper compartment was removed and emptied. The upper compartment was then immersed in 70% ethanol or methanol for a few minutes so that the glue melted and the filter became loose from the bottom of the syringe. This was picked up with a tweezer, taking care not to touch the rim and stain it.

Once fixed, it was mounted under a cover slip with the lower side of the filter facing up and examined for the presence of neutrophils that have crossed to the lower surface of the filter.[14,15]

### Phagocytosis of *P. gingivalis* Preparation of *P. gingivalis*

Commercially available *P. gingivalis* in blood agar was grown in schaedlers broth at 37°C, 48 hours prior to phagocytosis.[16] The cultures were spun at 1500 g for 10 minutes in a centrifuge. The deposits were washed twice with phosphate buffer saline and filtered twice through sterile gauze and resuspended in Hanks balanced salt solution at 5 × 10^7^ CFU/ml.

### Method

The contents of the tube were mixed and incubated at 37°C for 30 minutes. It was then centrifuged at 200 g for 5 minutes and the supernatant removed with Pasteur pipette leaving a droplet into which the sediment was resuspended. Smears were made, air dried, and stained with giemsa. At least 200 neutrophils were examined. A count of the number of ingested *P. gingivalis* associated with each cell was made.

### Bactericidal assay

To the above-mentioned contents, 4 ml of 0.01% methylene blue was added and centrifuged at 1500 g at 4°C for 10 minutes.[17] The supernatant was removed with Pasteur pipette leaving a droplet to resuspend the organisms. The suspension was incubated in an ice bath until ready for counting. Counting was done within 3 hours with a Neubaeurs chamber. At least 200 PMNL's/slide were counted. Dead bacteria appeared blue.

### Super oxide estimation

The solution was taken in a 10 ml beaker and illuminated for 10 minutes by keeping the beaker in an aluminium-foil-lined box fitted with a 15 W fluorescent lamp. Then the absorbance was read at 560 nm. One unit of super oxide activity was taken as that producing 50% inhibition of NBT reduction. The values obtained were subjected to statistical analysis. The test of significance applied was student unpaired *t* test.

### RESULTS

When the periodontal status of the diabetic group was compared with the healthy group, the amount of local factors and periodontal destruction was more in the healthy group, but some patients with diabetes showed comparatively more destruction than expected with lesser plaque scores.

The results of the present study also demonstrated that diabetic patients had defects in neutrophil functions when compared to healthy subjects as measured by chemotaxis, phagocytosis, microbicidal function, and super oxide released.

Table 1 compares the mean and standard deviation of chemotaxis of neutrophil between the two groups and it was seen that the diabetic group showed defective chemotaxis with a mean of 18.733 ± 5.663 and a mean of 30.667 ± 4.909 for the control group which was highly significant with the *P* value of 0.01.

**Table 1:** Mean and standard deviation of neutrophil chemotaxis for diabetics and controls

	Group	No.	Mean	Standard deviation	*t* value	*P* value
Neutrophil chemotaxis	Diabetic	30	18.733	5.663	0.1670	0.01***
	Control	30	30.667	4.909		

*** Highly significant

Table 2 compares the mean and standard deviation of phagocytosis of *P. gingivalis*as evaluated by mean particle number (MPN). It was seen that the MPN was significant in the healthy group with a mean of 3.600 ± 1.957 and a mean of 1.533 ± 0 .743 for the diabetic group with the *P* value of 0.05.

**Table 2:** Mean and standard deviation of phagocytosis of *P. gingivalis* for diabetics and controls

	Group	No.	Mean	Standard deviation	*t* value	*P* value
Phagocytosis of P. gingivalis	Diabetic	30	1.533	5.663	3.8240	0.05**
	Control	30	3.600	4.909		

** Significant

Table 3 assesses the intracellular killing capacity of neutrophils. It was revealed that the diabetic neutrophils had definitive low intracellular microbicidal function with a mean of 5.733 ± 2.987 for the diabetic group and a mean of 12.600 ± 8.798 for the control group with the *P* value of 0.01 which was again highly significant when compared to healthy subjects.

**Table 3:** Comparison of mean microbicidal activity for diabetics and controls

	Group	No.	Mean	Standard deviation	*t* value	*P* value
Microbicidal assay	Diabetic	30	5.733	2.987	2.8620	0.01***
	Control	30	12.600	8.798		

*** Highly Significant

Table 4 assesses the super oxide released by the neutrophils of the two groups as estimated by the activity of super-oxide dismutase. It was again seen that the super oxide released by the diabetic neutrophils was higher with a mean of 108.400 ± 15.697 when compared to the healthy neutrophils, which had a mean of 24.133 ± 14.212, as estimated by the intensity of the solution was more, which was very highly significant with the *P* value of 0.001.

**Table 4:** Comparison of mean and standard deviation of super oxide released between diabetics and controls

	Group	No.	Mean	Standard deviation	*t* value	*P* value
Super oxide release assay	Diabetic	30	108.400	15.697	15.4130	0.001****
	Control	30	24.133	14.212		

**** Very highly significant

## DISCUSSION

The probable influence of DM on the onset and duration of periodontal disease has been studied for many years. On the basis of experimental and clinical investigations, it is generally accepted that diabetes may result in a greater severity of periodontal disease. The mechanism of increased susceptibility to periodontitis in diabetics is not entirely clear.

According to a few investigators, the primary factor responsible for the development of diabetic complications is prolonged tissue response to hyperglycemia, which results in the production of advanced glycated end products. Also, there may be an increase in the local production of cytokines leading to connective tissue damage, bone resorption, and delayed wound repair.[1,18,19]

Several studies in diabetic animals and humans have reported defects in neutrophil functions, namely chemotaxis, phagocytosis, killing and microbicidal function, and super oxide released, which could be one of the reasons for increased susceptibility to periodontitis in these subjects.[2,9,20,21]

In the present study, two groups of subjects, 30 diabetics with periodontitis and 30 healthy individuals with periodontitis were compared for their periodontal status which included plaque index, gingival index, and attachment level, and neutrophil functions that included chemotaxis, phagocytosis of *P. gingivalis*, killing and microbicide of *P. gingivalis*, and amount of super oxide released.

When the periodontal status of healthy subjects with periodontitis was compared with that of the diabetic subjects with periodontitis, the amount of local factors and periodontal destruction was more in the healthy subjects, but some in the diabetic group showed comparatively more destruction than expected with lesser plaque scores. The mean plaque score was significantly less in the diabetic group than the healthy group with periodontitis who were age matched. This finding in our study is in agreement with Cohen *et al*.[7] who reported a significantly less plaque score in diabetics.

In the present study, in general, age of the patient and amount of local factors played an important role on periodontal status, and this is in agreement with studies done by Belting *et al*.[18]

Several investigators have stated that increased periodontitis is not detected in diabetic patients with plaque-free dentition, suggesting the presence and amount of local deposits is directly related to periodontal breakdown due to diminished host resistance.[22]

The results of the present study demonstrated that the diabetic patients with periodontitis had defects in neutrophil functions when compared to the healthy subjects with periodontitis. When chemotaxis was compared between the groups, defective chemotaxis was seen in the diabetic group, which is in agreement with the study done by Mowat and Baum[21] who showed significantly less chemotaxis with DM. They suggested that this defect in chemotaxis of diabetic leukocytes could contribute increased infections in these patients.

When phagocytosis of *P. gingivalis* was evaluated by MPN, that is the number of *P. gingivalis* phagocytosed by the neutrophils, it was seen that MPN was significantly higher in healthy subjects than the diabetic group. This result of our study is in agreement with the studies of Bybee *et al*[16] and Martha Walter *et al*,[23] who have reported impaired phagocytosis in diabetic individuals. In the present study, we used *P. gingivalis* as a model organism because it is one of the primary pathogens causing periodontitis.

When intracellular killing capacity of the neutrophils was assayed, it was seen that the diabetic PMNL's had a definite low intracellular microbicidal function. This abnormality was not only pronounced in diabetics but was also present to some extent in healthy subjects with periodontitis. This finding is in agreement with the study by Giovanni Salvi *et al*.[24] who stated that the local polysaccharides may alter oxidative burst capacity to impair killing.

When super oxide released was assayed using super oxide dismutase enzyme in the presence of nitroblue tetrazolium, it was found that the intensity of the blue color was more in diabetic samples than the healthy group. This suggested that more super oxide dismutase enzyme was required in the diabetic group to scavenge the free oxygen radicals. This result is in agreement with Martha Walters *et al.*[23] who found that the super oxide released by diabetic neutrophils was more than the healthy subjects with periodontitis.

In conclusion, when the neutrophil functions between diabetic patients and healthy subjects were compared, it was found that:

- Impaired neutrophil chemotaxis was observed in diabetic patients.
- Defective phagocytosis of *P. gingivalis* by neutrophils was observed in diabetic patients.
- The intracellular killing capacity of neutrophils was reduced in diabetic patients.
- The super oxide released by diabetic PMN's was drastically increased.

These above defects in the neutrophils of diabetic patients could be a possible mechanism to render them more susceptible to periodontal diseases.
